# The association between neighborhood deprivation and DNA methylation in an autopsy cohort

**DOI:** 10.18632/aging.205764

**Published:** 2024-04-24

**Authors:** Lindsay Pett, Zhenjiang Li, Sarina Abrishamcar, Kenyaita Hodge, Todd Everson, Grace Christensen, Marla Gearing, Michael S. Kobor, Chaini Konwar, Julia L. MacIsaac, Kristy Dever, Aliza P. Wingo, Allan Levey, James J. Lah, Thomas S. Wingo, Anke Hüls

**Affiliations:** 1 Department of Epidemiology, Rollins School of Public Health, Emory University, Atlanta, GA 30322, USA; 2 Gangarosa Department of Environmental Health, Rollins School of Public Health, Emory University, Atlanta, GA 30322, USA; 3 Department of Pathology and Laboratory Medicine, Emory University, Atlanta, GA 30322, USA; 4 Department of Neurology, Emory University School of Medicine, Atlanta, GA 30322, USA; 5 Department of Medical Genetics, University of British Columbia, Vancouver, BC, Canada; 6 BC Children’s Hospital Research Institute, Vancouver, BC, Canada; 7 Centre for Molecular Medicine and Therapeutics, Vancouver, BC, Canada; 8 Division of Mental Health, Atlanta VA Medical Center, Decatur, GA 30033, USA; 9 Department of Psychiatry, Emory University School of Medicine, Atlanta, GA 30322, USA; 10 Department of Human Genetics, Emory University, Atlanta, GA 30322, USA

**Keywords:** DNA methylation, brain tissue, neighborhood deprivation, neuropathology, epigenetics

## Abstract

Previous research has found that living in a disadvantaged neighborhood is associated with poor health outcomes. Living in disadvantaged neighborhoods may alter inflammation and immune response in the body, which could be reflected in epigenetic mechanisms such as DNA methylation (DNAm). We used robust linear regression models to conduct an epigenome-wide association study examining the association between neighborhood deprivation (Area Deprivation Index; ADI), and DNAm in brain tissue from 159 donors enrolled in the Emory Goizueta Alzheimer’s Disease Research Center (Georgia, USA). We found one CpG site (cg26514961, gene *PLXNC1*) significantly associated with ADI after controlling for covariates and multiple testing (p-value=5.0e^-8^). Effect modification by *APOE* ε4 was statistically significant for the top ten CpG sites from the EWAS of ADI, indicating that the observed associations between ADI and DNAm were mainly driven by donors who carried at least one *APOE* ε4 allele. Four of the top ten CpG sites showed a significant concordance between brain tissue and tissues that are easily accessible in living individuals (blood, buccal cells, saliva), including DNAm in cg26514961 (*PLXNC1*). Our study identified one CpG site (cg26514961, *PLXNC1* gene) that was significantly associated with neighborhood deprivation in brain tissue. *PLXNC1* is related to immune response, which may be one biological pathway how neighborhood conditions affect health. The concordance between brain and other tissues for our top CpG sites could make them potential candidates for biomarkers in living individuals.

## INTRODUCTION

Neighborhood socioeconomic status (SES) is complex and has unique social, cultural, physical, and economic attributes that can impact human health [1]. Residing in a deprived neighborhood has been associated with increased incidence of mental health conditions such as depression [2], increased risk of chronic conditions such as cardiovascular disease [2], and increased risk of brain-health diseases including Alzheimer’s disease [3, 4]. Research has demonstrated that living in a disadvantaged neighborhood can lead to chronic stress in the body, mainly through the immune and inflammatory response system [5]. The specific biological mechanisms that link neighborhood conditions to health outcomes are not fully understood.

A growing body of evidence suggests that epigenetics may help explain how neighborhood conditions impact health [6, 7]. DNA methylation (DNAm) is a well-studied epigenetic mechanism that involves the addition of a methyl group to DNA, typically at the 5-carbon of cytosine at cytosine-phosphate-guanine (CpG) dinucleotides, which can influence gene expression [8]. While the link between individual-level socioeconomic factors and differential DNAm has been well established [9–11], the effect of neighborhood-level socioeconomic factors on DNAm is less well known.

Existing studies on the relationship between neighborhood deprivation and DNAm are limited due to the novelty of the field of social epigenomics. Additionally, the implications of this relationship in the context of neuropsychiatric disorders are not well characterized. One study using blood samples and one study using saliva samples both found increased global DNAm among those living in more disadvantaged neighborhoods [12, 13]. Another study using blood samples identified three CpG sites that were associated with neighborhood deprivation, with one being linked to a gene (*MAOB*) that is related to Parkinson’s Disease [14]. Two other studies using blood samples found increased DNAm in nine genes related to stress and inflammation in the body [6, 15]. However, none of these identified CpG sites or genes were replicated across different studies. It is also important to note that none of these existing studies have examined the association between neighborhood deprivation and DNAm in brain tissue. DNAm changes in the brain specifically are important to study because they can provide indications of neuropathology outcomes such as Alzheimer’s disease (AD) [16–22] and depression [23, 24]. Many of these brain health outcomes have themselves been associated with neighborhood deprivation [2, 25, 26].

Given this gap in knowledge of how neighborhood deprivation impacts differential DNAm in the brain, we evaluated the association between the most established measure of neighborhood deprivation (Area Deprivation Index; ADI) and DNAm measured from brain tissue samples in a sample of mainly cognitively impaired, deceased donors from Georgia, USA, and analyzed whether those associations were independent of the observed AD neuropathology. DNAm at any CpG sites showing an association with ADI was further investigated in terms of their concordance across other (more accessible) tissues to explore their potential for serving as biomarkers in living individuals.

## RESULTS

### Description of study population

Our study included 159 donors. In the total study population, 89 (56.0%) were male, 142 (89.3%) were white, and the mean age at death was 76.6 years (SD 10.0) (Table 1). Of the total population, 56% had at least one *APOE* ε4 allele and 95.7% were clinically diagnosed with AD or some other form of dementia before death. Overall, 45.9% were classified as having the highest Braak Stage of 6, 69.2% were classified as having frequent CERAD, and 58.5% were classified as having a high ABC score. The mean ADI was 36.7 (SD 25.6), which is less deprived than the national average of ADI=50. Overall, 116 (73.0%) were classified into the lower ADI group (ADI<50; less deprived). Compared to those in the high ADI group (ADI≥50; more deprived), those in the low ADI group were more likely to be white (95.7% vs. 72.1% in the high ADI group) and have at least a college degree (79.3% vs. 72.1% in the high ADI group), with the two groups being similar in other demographic categories. Additionally, those in the low ADI group were more likely to be diagnosed with AD or some other form of dementia (97.4.1% vs. 90.7% in the high ADI group) but were similar on other clinical categories including Braak Stage, CERAD, ABC score, and *APOE* ε4 alleles. The study characteristics of our analysis sample did not significantly differ from the full cohort (Supplementary Table 8).

**Table 1:** Characteristics of individuals from the ADRC cohort, stratified by Area Deprivation Index group (low=ADI<50 corresponding to less deprived than the national average), (high=ADI ≥50 corresponding to more deprived than the national average).

**Sample characteristic n (%) or mean [SD]**	**Total (n=159)**		**Low ADI (n=116)**		**High ADI (n=43)**	
ADI^1^	36.7	[25.6]	24.1	[15.5]	70.5	[14.1]
**Demographics**						
Race						
White	142	(89.3)	111	(95.7)	31	(72.1)
Black	17	(10.7)	5	(4.3)	12	(27.9)
Sex						
Male	89	(56.0)	63	(54.3)	26	(60.5)
Female	70	(44.0)	53	(45.7)	17	(39.5)
Age at death	76.6	[10.0]	76.6	[9.6]	76.6	[11.1]
Education attainment						
High school or less	36	(22.6)	24	(20.7)	12	(27.9)
College degree	76	(47.8)	56	(48.3)	20	(46.5)
Graduate degree	47	(29.6)	36	(31.0)	11	(25.6)
**Clinical variables**						
Braak Stage						
Stage 1	16	(10.1)	11	(9.5)	5	(11.6)
Stage 2	11	(6.9)	6	(5.2)	5	(11.6)
Stage 3	20	(12.6)	17	(14.7)	3	(7.0)
Stage 4	17	(10.7)	12	(10.3)	5	(11.6)
Stage 5	22	(13.8)	18	(15.5)	4	(9.3)
Stage 6	73	(45.9)	52	(44.8)	21	(48.8)
CERAD^2^						
No	35	(22.0)	26	(22.4)	9	(20.9)
Sparse	4	(2.5)	4	(3.4)	0	(0.0)
Moderate	10	(6.3)	7	(6.0)	3	(7.0)
Frequent	110	(69.2)	79	(68.1)	31	(72.1)
ABC^3^						
Not	15	(9.4)	10	(8.6)	5	(11.6)
Low	29	(18.2)	21	(18.1)	8	(18.6)
Intermediate	22	(13.8)	17	(14.7)	5	(11.6)
High	93	(58.5)	68	(58.6)	25	(58.1)
*APOE^4^* ε4 Allele(s)						
0	70	(44.0)	51	(44.0)	19	(44.2)
1	68	(42.8)	53	(45.7)	15	(34.9)
2	21	(13.2)	12	(10.3)	9	(20.9)
Cognitive classification						
No dementia	7	(4.4)	3	(2.6)	4	(9.3)
Other dementia	66	(41.5)	51	(44.0)	15	(34.9)
AD^5^	86	(54.2)	62	(53.4)	24	(55.8)

^1^Area Deprivation Index; ^2^Consortium to establish a register for AD; ^3^Amyloid, Braak and CERAD; ^4^Apolipoprotein E; ^5^Alzheimer’s disease.

### Association between neighborhood deprivation and DNA methylation in the brain

**Figure 1:**
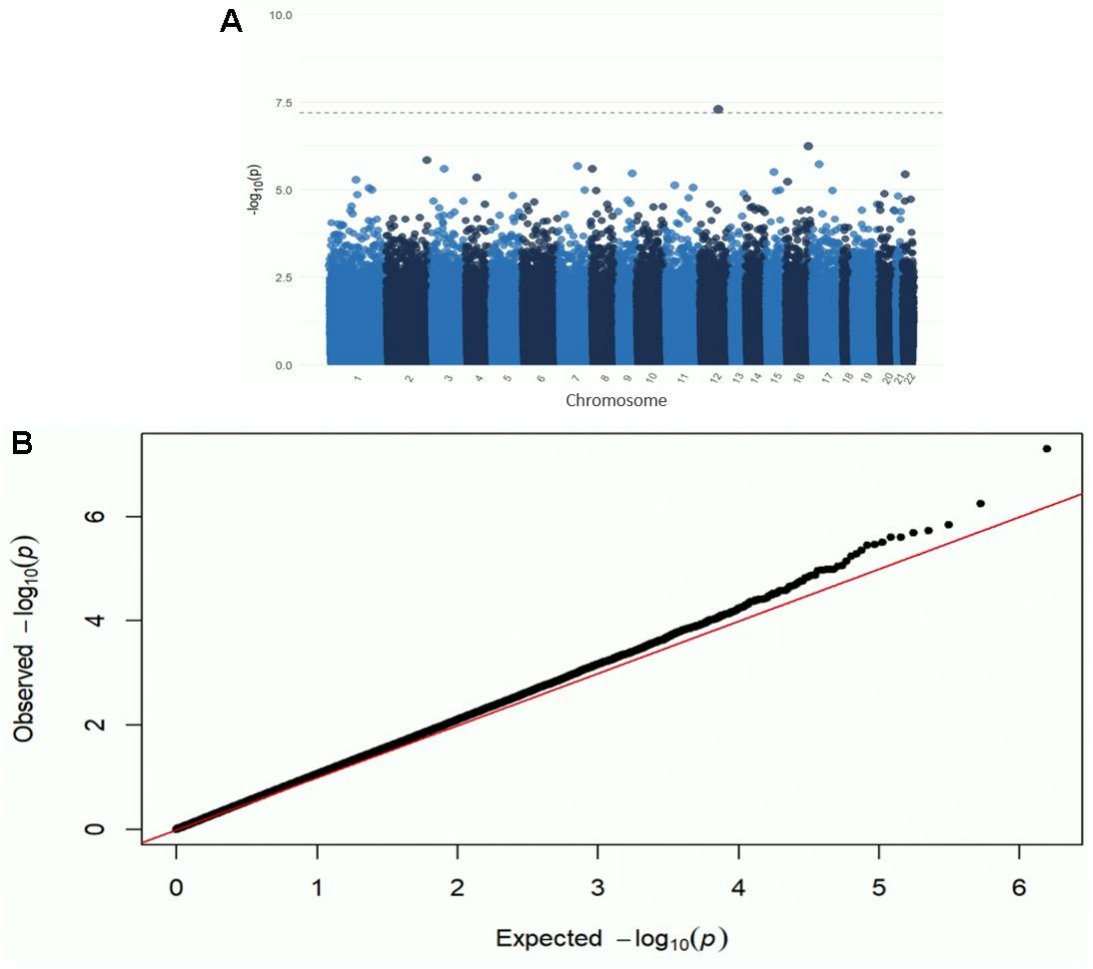
Manhattan (**A**) and QQ plot (**B**) from the EWAS of DNAm with the ADI. Adjusted for race, sex, educational attainment, age at death, APOE genotype, cell type, and post-mortem interval. Bonferroni-threshold: 0.05/789889 = 6.33e^-8^ (λ=0.94).

**Figure 2:**
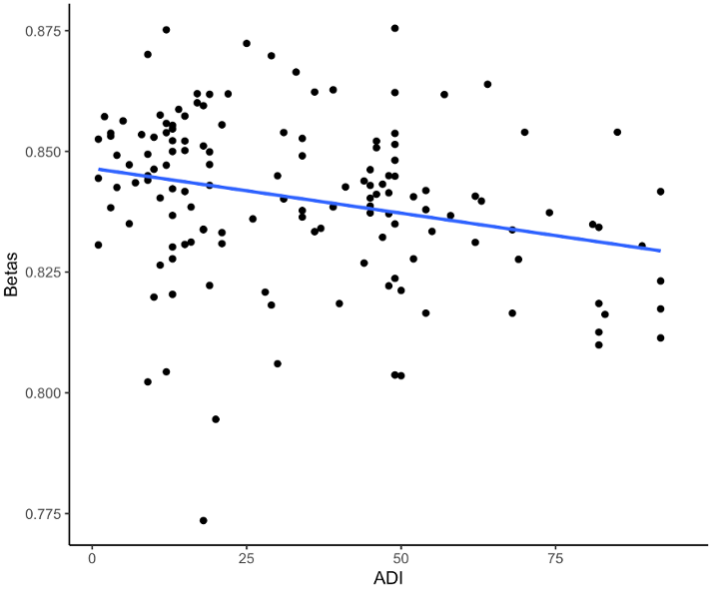
**Scatterplot of DNAm beta values and the ADI from the EWAS of DNAm with the ADI for the CpG site cg26514961 (*PLXNC1*).** The dots represent the DNAm beta and ADI values for a participant, and the blue line represents the (unadjusted) linear relationship between the DNAm beta values and the ADI.

One CpG site (cg26514961, gene *PLXNC1*) was significantly associated with ADI when controlling for self-reported race, sex, *APOE* ε4, education, age at death, cell type proportions, and post-mortem interval (p-value=5.0e^-8^) (Figures 1, 2 and Table 2). A 20-unit increase in ADI was associated with a -0.0052 decrease in DNAm beta value (Table 2). No other CpG sites were significantly associated with ADI (Figure 1). The other top nine CpG sites and their associated genes were cg08087060 (*KLHDC4*), cg01291468 (*UGT1A10*, *UGT1A7*, *UGT1A9*, and *UGT1A8*), cg16241648 (*ARPC1A*), cg20912923 (a *CSMD1*), cg09431774 (*KIAA1671*) and the intergenic CpG sites cg05419854, cg15953452, cg06787422, and cg13521319 (Table 2 and Supplementary Figure 2). The epigenome-wide summary statistics are available online (Supplementary EWAS Output.xlsx). Similar results were found after additional adjustment for neuropathology markers of AD (CERAD, Braak Stage, and ABC); thus indicating that these results were independent of the degree of neuropathology (Supplementary Table 2). Results were also similar after excluding the 2.5% cognitively normal donors from the EWAS (Supplementary Table 7). Our regional analysis using DMRs did not find any regions to be statistically significant. The top ten regions are summarized in Supplementary Table 10.

**Table 2:** Top ten CpG sites from the epigenome-wide association study of DNA methylation with the Area Deprivation Index, stratified by the presence or absence of the *APOE* ε4 allele.

				**Total (n=159)**		**ε4 present (n=89)**		**ε4 absent (n=70)**		**Effect modification**
**CpG**	**Chromosome**	**Position**	**Gene(s)**	**Effect estimate**	**P-value**	**Effect estimate**	**P-value**	**Effect estimate**	**P-value**	**P-value**
cg26514961	12	94566784	*PLXNC1*	**-0.0052**	**5.0e^-8^**	-0.0050	0.0033	-0.0008	0.0002	8.7e^-6^
cg08087060	16	87795808	*KLHDC4*	-0.0040	5.7e^-7^	-0.0036	0.0030	-0.0007	0.0009	4.6e^-5^
cg01291468	2	234589374	*UGT1A10;UGT1A7;* *UGT1A9;UGT1A8*	0.0034	1.4e^-6^	0.0027	0.0098	0.0004	0.0019	0.0003
cg05419854	17	19398395	*-*	-0.0058	1.8e^-6^	-0.0042	0.0190	-0.0008	0.0004	1.0e^-5^
cg16241648	7	98923114	*ARPC1A*	0.0016	2.1e^-6^	0.0018	0.0046	0.0003	0.0020	0.0002
cg20912923	8	2885516	*CSMD1*	-0.0026	2.5e^-6^	-0.0021	0.0083	-0.0002	0.0008	3.1e^-5^
cg15953452	3	63053400	*-*	-0.0050	2.5e^-6^	-0.0042	0.0105	0.0008	0.0001	6.2e^-6^
cg06787422	15	63331851	*-*	-0.0024	3.1e^-6^	-0.0015	0.0411	-0.0002	0.0012	0.0001
cg13521319	9	133423844	*-*	-0.0018	3.4e^-6^	-0.0020	0.0019	-0.0001	0.0046	0.0007
cg09431774	22	25465561	*KIAA1671*	-0.0028	3.6e^-6^	-0.0020	0.0249	-0.0002	0.0002	8.6e^-6^

**Bold:** statistically significant at the Bonferroni threshold of 6.33e-8.

Effect estimates can be interpreted per a 20-unit increase in ADI. All models were adjusted for the following covariates: race, sex, educational attainment, age at death, apolipoprotein E (*APOE*) genotype, cell type, and post-mortem interval.

Next, we investigated whether the associations with the top ten CpG sites from the EWAS of ADI were modified by *APOE* ε4 allele. We found nominally significant (p-value < 0.05) effect modifications by presence versus absence of the *APOE* ε4 allele for all our top ten CpG sites. Effect estimates for associations between the ADI and DNAm observed in the whole study population were similar as the estimates observed among donors with at least one *APOE* ε4 allele. Effect estimates were alleviated toward the null among donors without any *APOE* ε4 alleles. No CpG sites were found to be significantly associated with ADI in either *APOE* ε4 group (Supplementary Figure 1A, 1B).

We then examined whether any of the top ten CpG sites from the EWAS of ADI were associated with AD pathology (CERAD, Braak Stage, and ABC). None of the ten CpG sites were significantly associated with any of the three neuropathology outcomes (Table 3).

**Table 3:** Association between the top ten CpG sites from the epigenome-wide association study of Area Deprivation Index (compare Table 2), and their association with neuropathology markers (CERAD, ABC and Braak stage).

				**CERAD^1^**	**ABC^2^**	**Braak stage**
**CpG**	**Chromosome**	**Position**	**Gene(s)**	**Effect estimate (95%-CI)**	**Effect estimate (95%-CI)**	**Effect estimate (95%-CI)**
cg26514961	12	94566784	*PLXNC1*	0.41 (-0.85, 1.67)	0.67 (-0.35, 1.70)	0.41 (-0.78, 2.56)
cg08087060	16	87795808	*KLHDC4*	0.57 (-1.17, 2.32)	0.08 (-1.47, 1.63)	0.57 (-2.73, 1.92)
cg01291468	2	234589374	*UGT1A10;UGT1A7;* *UGT1A9;UGT1A8*	-1.20 (-3.50, 1.09)	-1.14 (-3.13, 0.82)	-1.20 (-4.67, 1.49)
cg05419854	17	19398395	*-*	-0.89 (-2.17, 0.37)	-0.39 (1.50, 0.71)	-0.89 (-1.05, 2.52)
cg16241648	7	98923114	*ARPC1A*	0.93 (-3.42, 5.30)	-1.08 (-4.96, 2.77)	0.93 (-6.89, 5.12)
cg20912923	8	2885516	*CSMD1*	0.46 (-2.25, 3.18)	1.54 (-0.66, 3.77)	0.46 (-1.82, 5.46)
cg15953452	3	63053400	*-*	0.11 (-1.33, 1.55)	0.56 (-0.62, 1.75)	0.11 (-0.66, 2.93)
cg06787422	15	63331851	*-*	-0.13 (-3.33, 3.06)	0.50 (-2.22, 3.22)	-0.13 (-3.20, 4.94)
cg13521319	9	133423844	*-*	-1.37 (-5.24, 2.46)	-0.69 (-4.24, 2.84)	-1.37 (-6.76, 4.03)
cg09431774	22	25465561	*KIAA1671*	0.37 (-2.17, 2.91)	-0.10 (-2.41, 2.21)	0.37 (-4.31, 2.78)

^1^Consortium to Establish a Register for AD; ^2^Amyloid, Braak, and CERAD.

Effect estimates can be interpreted per a 0.1-unit increase in DNAm. All models were adjusted for the following covariates: race, sex, educational attainment, age at death, apolipoprotein E (*APOE*) genotype, cell type, and post-mortem interval.

### Look-up of top hits in mQTL or cross-tissue databases

Genetic variants influence DNAm patterns, so we investigated whether the identified DNAm associations were likely driven by genetic variant effects (mQTLs). According to the GoDMC database [27], of the top ten CpG sites from the EWAS of ADI, three were associated with at least one mQTL, namely cg26514961 (*PLXNC1*), cg01291468 (*UGT1A10*, *UGT1A7*, *UGT1A9*, and *UGT1A8*), and cg06787422 (intergenic) (Supplementary Table 3). To evaluate the correlation of DNAm at our top ten CpG sites across different tissues, we used the BECon tool and Gene Expression Omnibus Database [Accession code GSE111165]. Two CpG sites (cg20912923 (*CSMD1*) and cg06787422 (intergenic)) exhibited blood–brain concordance using the BECon tool (Supplementary Table 4A). Both of these sites exhibited 75-90% percentile mean correlations between blood and brain samples. Using the Gene Expression Omnibus Database, only cg15953452 (intergenic) exhibited significant blood-brain concordance (Supplementary Table 4B). Two CpG sites, cg26514961 (*PLXNC1*) and cg16241648 (*ARPC1A)*, exhibited significant buccal cell-brain concordance. Four CpG sites (cg26514961 (*PLXNC1*), cg16241648 (*ARPC1A)*, cg15953452 (intergenic), and cg06787422 (intergenic)) exhibited significant saliva-brain concordance.

### Pathway enrichment analysis

To further aid the interpretation of our top associations, we performed a gene ontology (GO) and KEGG pathway enrichment analysis based on the top 1000 CpG sites with lowest raw p-values. After correction for multiple testing (FDR <0.05), we did not identify any GO terms or KEGG pathways with an overrepresentation of genes containing significantly, differentially methylated CpGs that would indicate an enriched biological pathway. GO terms and KEGG pathways that were nominally significant (raw p<0.05) are included in the supplement (Supplementary Table 5A, 5B).

## DISCUSSION

In the ADRC autopsy cohort of 159 donors, we found one CpG site (cg26514961, gene *PLXNC1*) that was significantly associated with the ADI in brain tissue after controlling for covariates and multiple testing. Effect modification by *APOE* ε4 was found to be statistically significant for the top ten CpG sites from the EWAS, indicating that the observed associations between ADI and DNAm were mainly driven by donors who carried at least one *APOE* ε4 allele. Four of the top ten CpG sites showed a significant concordance between brain tissue and tissues that are easily accessible in living individuals (blood, buccal cells, saliva), including DNAm in cg26514961 (*PLXNC1*). This suggests that differential DNAm in these CpG sites could potentially be detected prior to death. None of the top ten CpG sites from the EWAS of ADI were associated with AD pathology in this autopsy cohort and the EWAS results were robust to additional adjustment for neuropathology markers. This indicates that the identified associations between ADI and differential DNAm in the brain were independent of the degree of AD-related neuropathology.

The EWAS identified cg26514961 as being significantly associated with the ADI, which is associated with the *PLXNC1* gene. This gene is believed to be related to the immune response in the body [28]. Additionally, the corresponding RNA and protein levels are altered in the brains of people with AD [29]. The protein that this gene encodes regulates melanocyte adhesion, and viral semaphorins are thought to modulate the immune response through binding to this receptor [29]. Previous research has suggested the immune response as a potential biological pathway of how neighborhood deprivation affects the body [5]. This hypothesis is further supported by three additional genes that were among the top three CpG sites (cg01291468 [*UGT1A7*, *UGT1A8*, and *UGT1A9*]*)* and which have all been linked to immunosuppression [30–32]; thus providing further evidence that neighborhood deprivation impacts health through the immune response. Two additional genes among our top ten CpG sites have been associated with brain-related health outcomes and aging. *KLHDC4* (cg08087060) is associated with Huntington’s disease [33], and *CSMD1* (cg20912923) is related to learning and memory [34]. In a meta-analysis of brain tissue-based EWAS in Alzheimer’s disease (n=1453), our top ten CpG sites were not found in any of the studies the authors examined, and none of the top 25 CpG sites they found to be statistically significant for Alzheimer’s disease were associated with ADI in our analysis (Supplementary Table 9) [35].

Our study found concordance between brain and other tissues in four of our top ten CpG sites. It is important to examine the concordance between brain tissue and other tissues (such as blood, saliva, and buccal) because brain tissue samples are not accessible from living donors, whereas these three other tissues are. Differential DNAm in tissues that are easily accessible in living individuals can serve as biomarkers of exposures or to predict related health outcomes. Thus, if DNAm profiles in brain tissue are correlated with other tissues, those profiles can potentially be used to identify individuals at heightened risk, and may lead to earlier access to preventative care.

None of the top ten CpG sites have been identified in prior studies as being related to DNAm and ADI, most likely due to the different tissues that were used. Two prior studies found increased global DNAm among those living in more disadvantaged neighborhoods [12, 13]. These studies did not examine particular CpG sites or genes, so it is unclear which locations experienced increased or decreased DNAm levels. Another study found three CpG sites that were associated with neighborhood deprivation, with one being linked to a gene that is related to Parkinson’s Disease [14]. None of these three CpG sites were identified in our EWAS (Supplementary Table 6), but it is of note that their study also identified genes associated with an aging-related disease. Two other studies found increased DNAm in genes related to stress and inflammation in the body [6, 15], which is closely linked to the immune response pathway that two of our top ten CpG sites were linked to [36]. Overall, our findings related to stress and inflammation align with pathways identified in previous research, but more studies are needed to replicate our findings and to identify other CpG sites and genes which are related to neighborhood deprivation.

We found evidence of effect modification by *APOE* ε4 in the EWAS of ADI, indicating that the observed associations between ADI and DNAm were mainly driven by donors who carried at least one *APOE* ε4 allele. This aligns with previous research, which suggests that there are differences in epigenome-wide methylation among *APOE* ε4 carriers and non-carriers in blood samples in many genetic positions and loci [37]. Further research is needed to investigate how DNAm differs by *APOE* ε4 being present or absent, especially in brain tissue.

None of the top ten CpG sites from the EWAS of ADI were associated with AD pathology in this autopsy cohort. This finding could be due to most participants in our sample being cognitively impaired, which limits the statistical power to detect differences between impaired and non-impaired individuals. More research on this association with a larger sample of non-impaired individuals is needed to better understand the relationship between these CpG sites and AD.

Lastly, three of our top ten CpG sites were associated with at least one known mQTL, which is an indicator of the genetic influence on DNAm levels [27]. While we are unable to disambiguate the effects of the environment and genes on DNAm levels, only a proportion of the variation in DNAm levels is explained by genetic effects. In fact, the joint effects of environmental factors and single nucleotide polymorphisms (SNP) have been found to be larger contributors to DNAm variation than SNPs alone [38].

Our study has several strengths. One major strength is that our study is the first known study on the association between neighborhood deprivation and DNAm in brain tissue, which is difficult to obtain and the most relevant tissue to study brain-related health outcomes. Studying DNAm changes in brain tissue is especially important because it can provide insight into neuropathology outcomes such as Alzheimer’s disease [16–22] and depression [23, 24]. These brain health outcomes have themselves been associated with neighborhood deprivation [2, 25, 26], which is the reason more research on neighborhood deprivation using brain tissue is needed. Another strength of our study is that we had diversity of neighborhood deprivation. In our study, the ADI ranged from 1 to 95, thus including very deprived and very privileged neighborhoods. Another strength of our study is that we used the Infinium methylation EPIC array as opposed to the Illumina Infinium HumanMethylation450 (450K) BeadChip array. The EPIC array covers more than 850,000 methylation sites whereas the 450K array only covers 450,000 methylation sites. Only one of the five previous studies on the association between neighborhood deprivation and DNAm used the EPIC array [13]. Of the top ten CpG sites associated with ADI in our cohort, four CpG sites were only available on the EPIC array.

Our study has a few limitations. Our sample size was relatively small (n=159), which limited the statistical power to detect associations. Additionally, our sample was not racially diverse and only contained self-reported White and Black donors. Only 10.7% of participants in our sample were Black, limiting our ability to detect racial differences. Thus, we are unable to generalize our results to other racial or ethnic groups. Another limitation of our study is that we only had information on the donors’ last known address. It is possible that the donors moved around a lot during their life, or only moved to their last address at the end of their life. In these cases, the long-term or even life-term exposure to neighborhood deprivation would not be captured in the data. It is possible that the neighborhood conditions of where someone grew up or lived during most of their life are more relevant to studying the association with DNA methylation as opposed to where they lived at the end of their life, but further research is needed to elucidate these effects throughout the lifespan. Another limitation of our study is that the 2020 ADI measure we used does not correspond with the donors’ years of death. This could lead to measurement error in our study, which may result in biased estimates. A final limitation of our study is that very few participants were not cognitively impaired (2.5%). Because the majority of participants had some form of cognitive impairment, the statistical power to detect differences between impaired and non-impaired participants was rather limited. Furthermore, most participants exhibited Braak Stage 6 (45.9%), had frequent CERAD (69.2%), and had a high ABC score (58.5%). These are extreme values as compared to the general US population, demonstrating that our population was not representative of the larger US or Georgia population.

Overall, our study identified one CpG site (cg26514961, *PLXNC1* gene) that was significantly associated with neighborhood deprivation in brain tissue. We also found evidence of effect modification by *APOE* ε4, suggesting that the observed associations between ADI and DNAm were mainly driven by donors who carried at least one *APOE* ε4 allele. Our study provides motivation to conduct larger studies on the association between neighborhood deprivation and DNAm in the brain to replicate and expand upon our findings. The identification of significant CpG sites could provide novel insights into the etiology of health disparities, and the concordance between brain and other tissues for our top CpG sites could make them potential candidates for biomarkers in living individuals.

## MATERIALS AND METHODS

### Study population

The study population was derived from brain tissue donors recruited by the Emory Goizueta Alzheimer’s Disease Research Center (ADRC). Most of the donors in this study were patients diagnosed as having Alzheimer’s Disease and were treated at the Emory Clinic or Emory University Hospital. In total, 1011 donors enrolled in the study until the third quarter of 2020 (Supplementary Table 1). The inclusion criteria for our study were the following: 1) residential addresses within Georgia; 2) age at death of at least 55; 3) died after 1999; 4) no missing values in outcomes and key covariates which include race, sex, educational attainment, *APOE* genotype; 5) DNAm data was available. Based on these criteria, 159 donors remained in the analysis. Written consent for brain donation was obtained from next of kin as required under Georgia law. Emory University’s Institutional Review Board approved this study.

### Assessment of neighborhood deprivation

Neighborhood deprivation was defined using the Area Deprivation Index (ADI), a census-based socioeconomic index developed by Kind et al. [39]. The ADI is calculated using socioeconomic status domains of income, education, employment, and housing quality indicators obtained from the American Community Survey. Using these domains, the ADI is calculated from 17 census indicators that are multiplied by previously published factor weights and summed for each census block group and then transformed into a standardized index [20]. The ADI assigns ranked percentiles that range from 1 to 100, where 100 represents the most deprived neighborhood. A neighborhood is defined as a census block group, which is the smallest geographic unit used by the United States Census Bureau to tabulate 100-percent data. A census block group comprises a set of blocks that generally contain 600 to 3000 people and is the smallest unit with detailed demographic-economic characteristics [40]. We linked the 2020 ADI to each participant’s geocoded residential address at the time of their death using Federal Information Processing Standards codes [41].

### Assessment of neuropathologic markers

The ADRC conducted neuropathologic evaluations on every donor’s brain using diagnostic criteria and established research evaluations. The neuropathologic assessments evaluated the severity of AD-related neuropathology changes, which included a variety of stains and immunohistochemical preparations as well as semi-quantitative scoring of multiple neuropathologic changes in brain regions by experienced neuropathologists using published criteria. AD neuropathology was assessed using the Consortium to Establish a Register for AD (CERAD) score, Braak stage, and a combination of Amyloid, Braak, and CERAD (ABC) score. CERAD score represents the prevalence of neuritic plaques with four levels from zero neuritic plaques to frequent. Braak stage is a staging scheme which represents neurofibrillary tangles (NFTs) and has six stages (Stage I-VI), with higher stages indicating a wider distribution of NFTs in the brain. ABC score combines CERAD and Braak Stage with the prevalence of Amyloid plaques and is converted to one of four levels of AD neuropathologic changes: not, low, intermediate, or high.

### Assessment of DNA methylation

Fresh, frozen prefrontal cortex samples were collected from donors at autopsy, and DNA was isolated from these samples using the QIAGEN GenePure kit. Illumina Infinium HumanMethylationEPIC BeadChips arrays were used to assess DNAm in the 159 samples and 6 replicates for quality control to assess the background technical variation (root mean square error (RMSE) ranged from 0.022-0.028). We followed a validated quality control and normalization pipeline as previously published [42]. Pre-processing and statistics were completed using R (v4.2.0). All DNAm data were preprocessed to identify low-quality samples, exclude specific probes, and reduce the impact of batch effects. Raw intensity files were converted to methylation beta values ranging on a continuous scale from 0 to 1 for each of the CpG sites measured on the array. The Illumina’s 636 control probes were used via the R package ewastools to assess technique parameters including array staining, extension, hybridization, target removal, specificity, and bisulfite conversion [43]. Additional sample outlier detection was implemented based on detection *p*-value, beadcount, and distance from the group average in principal components. The Funnorm function and Combat function were used to normalize the distributions to reduce technical variation and correct for differences between type I and type II probe signals. The following probes were further removed: XY probes, low-quality probes with missing in more than 5% of samples, probes with poor detection p-values, probes predicted to cross-hybridize, probes that bind to the sex chromosomes, polymorphic probes, and probes with infinite values. In total, after all preprocessing steps, 159 samples and 789,286 CpG sites remained for the down-stream analysis. We used the estimateCellCounts function in the R package minfi to obtain the cell-type proportions (neuronal vs. non-neuronal cells) for each sample using the most recent prefrontal cortex database [44, 45].

### Confounder assessment

Confounders were identified based on existing literature. All models were adjusted for the following covariates: race, sex, educational attainment, age at death, apolipoprotein E (*APOE*) genotype, cell type, and post-mortem interval. Due to the sample only containing White and Black participants, the race variable was binary. Educational attainment was defined as the highest level of education completed by the participant and classified into high school or less, college degree, and graduate degree. *APOE* genotype had three levels in the analysis: no ε4 allele, single ε4 allele, and double ε4 allele. The *APOE ε4* allele is a well-known risk factor of developing Alzheimer’s disease, and the current analysis considered: 0, 1, and 2 *ε4* alleles. Also, a binary *APOE* genotype (ε4 absent vs. present) was used for testing the effect modification by the genotype. Binary *APOE* genotype was used for effect modification analyses to conserve statistical power in analyses (see Table 1 for a distribution of *APOE ε4* genotypes).

### Statistical analysis

To identify DNAm patterns in brain tissue that are associated with ADI, we conducted an epigenome-wide association study (EWAS) of single CpG sites and an analysis of differentially methylated regions (DMRs). For the EWAS, we ran a robust linear regression model using the RLM function within the MASS package with ADI as the independent variable and DNAm beta values at each CpG site as a dependent variable, adjusting for self-reported race, sex, *APOE* genotype, education, age at death, cell type, and post-mortem interval. We applied a Bonferroni threshold to correct for multiple testing based on the number of tested CpG sites (threshold: 0.05/789889 = 6.33e^-8^). Associations between ADI and DMRs were analyzed using the R package dmrff.

We conducted several sensitivity analyses to evaluate the robustness of our EWAS findings. First, we adjusted for neuropathology markers (CERAD, Braak Stage, and ABC) to investigate whether the identified associations were independent of the degree of neuropathology. Second, we conducted an EWAS of ADI after excluding the 2.5% cognitively normal donors. Third, since *APOE* ε4 is a well-known risk factor for developing AD, we included a multiplicative interaction term between ADI and *APOE* genotype (presence or absence of ε4 allele) in our EWAS to test for effect modification and presented the stratified effect estimates derived from that interaction model.

Next, we investigated whether DNAm patterns in brain tissue that are associated with ADI are also linked with neuropathology markers. We ran linear regression models using each of the top ten CpGs as the independent variables, and three neuropathology outcomes (CERAD, Braak Stage, and ABC) as dependent variables in separate models, adjusting for ADI, self-reported race, sex, *APOE* genotype, education, age at death, cell type, and post-mortem interval.

We conducted additional analyses for the top ten CpG sites in the EWAS analysis to evaluate their correlation across different tissues and how methylation at those sites is affected by genotypic variation. This included blood–brain concordance analysis using the Blood–Brain Epigenetic Concordance (BECon) tool [46], blood-brain, buccal-brain, and saliva-brain concordance using the data from Braun et al. (2019) on the Gene Expression Omnibus Database [Accession code GSE111165] [47, 48] and methylation quantitative trait loci (mQTL) mapping using the GoDMC database [27]. To further aid the interpretation of our top associations, we conducted a gene ontology (GO) and KEGG pathway enrichment analysis which was a look-up of top hits in mQTL and cross-tissue databases using the R package missMethyl based on the top 1000 CpG sites with lowest raw p-values [49].

## Supplementary Material

Supplementary Figures

Supplementary Tables 1-3

Supplementary Table 4

Supplementary Tables 5-10

Supplementary EWAS Output
