# Supplementary material for: The association between neighborhood deprivation and DNA methylation in an autopsy cohort
Source: Aging (Albany NY). 2024 Apr 24;16(8):6694–716. doi: 10.18632/aging.205764 (PMC11087100; doi:10.18632/aging.205764)
Supplement: Supplementary Figures [file aging-16-205764-s001.pdf]

SUPPLEMENTARY FIGURES

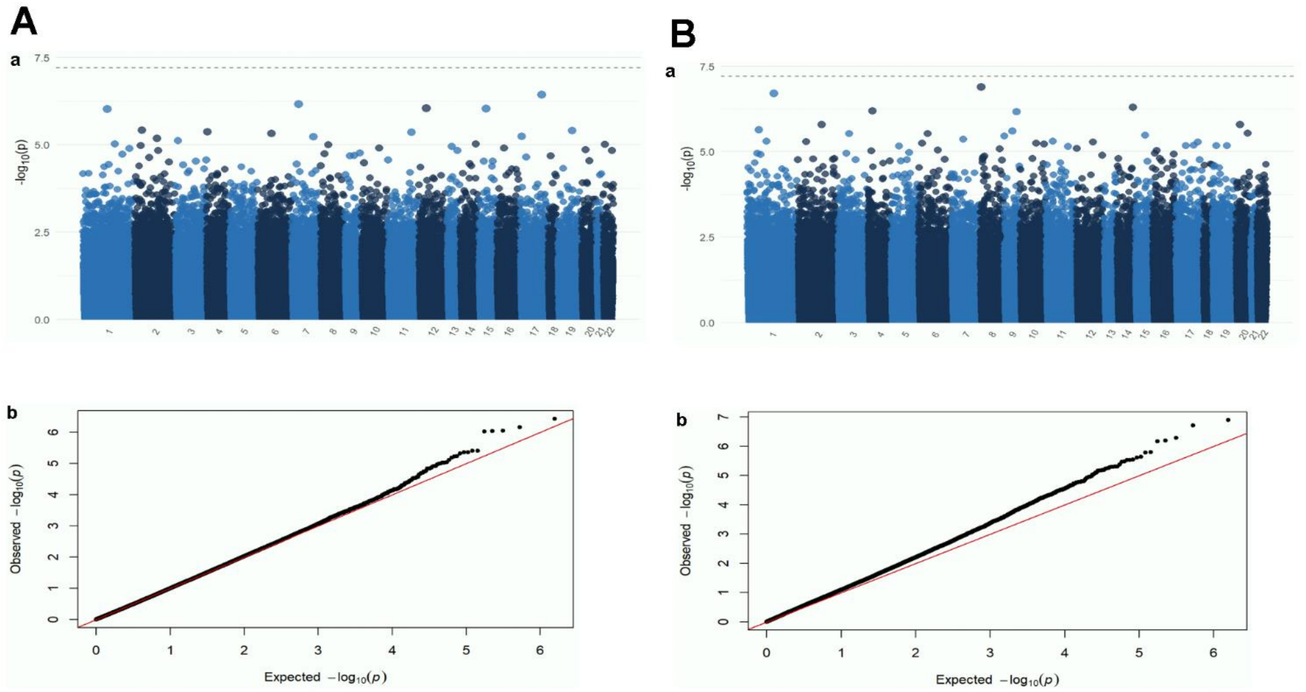

Supplementary Figure 1. (**A**) Manhattan (**a**) and QQ plot (**b**) from the EWAS of ADI in APOE ε4 present participants. Adjusted for race, sex, educational attainment, age at death, cell type, and post-mortem interval. Bonferroni-threshold: 0.05/789889 = 6.33e-8 (λ=0.94). (**B**) Manhattan (**a**) and QQ plot (**b**) from the EWAS of ADI in APOE ε4 absent participants. Adjusted for race, sex, educational attainment, age at death, cell type, and post-mortem interval. Bonferroni-threshold: 0.05/789889 = 6.33e-8 (λ=0.94).

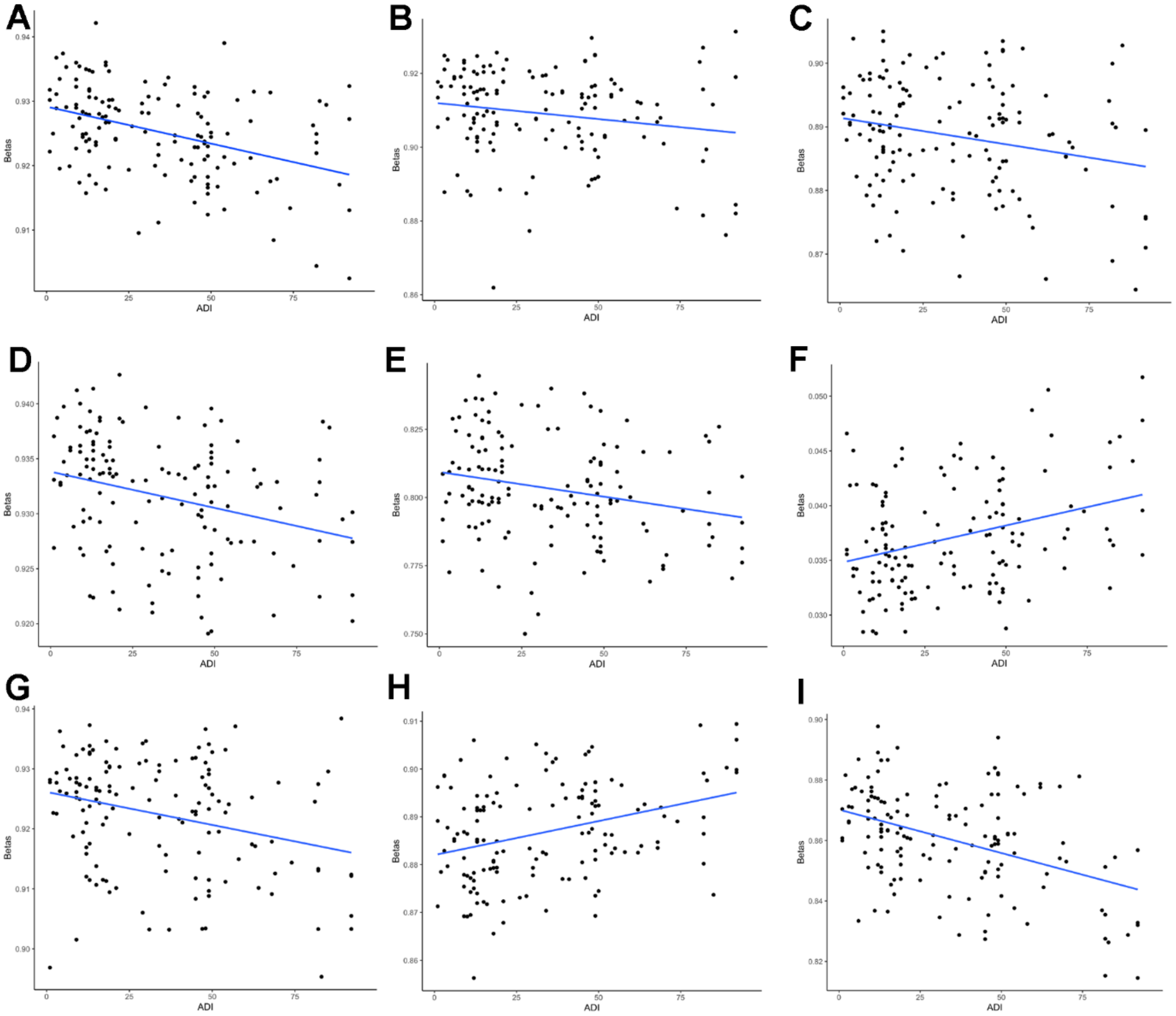

Supplementary Figures 2. Scatterplots of beta values and the ADI from the EWAS of DNAm with the ADI for the top ten CpG sites from the EWAS of ADI for the total study population. The dots represent the DNAm beta and ADI values for a participant, and the blue line represents the linear relationship between the DNAm beta values and the ADI. (**A**) Scatterplot of beta values and the ADI from the EWAS of DNAm with the ADI for cg08087060 from the EWAS of ADI for the total study population. The dots represent the DNAm beta and ADI values for a participant, and the blue line represents the linear relationship between the DNAm beta values and the ADI. (**B**) Scatterplot of beta values and the ADI from the EWAS of DNAm with the ADI for cg01291468 from the EWAS of ADI for the total study population. The dots represent the DNAm beta and ADI values for a participant, and the blue line represents the linear relationship between the DNAm beta values and the ADI. (**C**) Scatterplot of beta values and the ADI from the EWAS of DNAm with the ADI for cg05419854 from the EWAS of ADI for the total study population. The dots represent the DNAm beta and ADI values for a participant, and the blue line represents the linear relationship between the DNAm beta values and the ADI. (**D**) Scatterplot of beta values and the ADI from the EWAS of DNAm with the ADI for cg16241648 from the EWAS of ADI for the total study population. The dots represent the DNAm beta and ADI values for a participant, and the blue line represents the linear relationship between the DNAm beta values and the ADI. (**E**) Scatterplot of beta values and the ADI from the EWAS of DNAm with the ADI for cg20912923 from the EWAS of ADI for the total study population. The dots represent the DNAm beta and ADI values for a participant, and the blue line represents the linear relationship between the DNAm beta values and the ADI. (**F**) Scatterplot of beta values and the ADI from the EWAS of DNAm with the ADI for cg15953452 from the EWAS of ADI for the total study population. The dots represent the DNAm beta and ADI values for a participant, and the blue line represents the linear relationship between the DNAm beta values and the ADI. (**G**) Scatterplot of beta values and the ADI from the EWAS of DNAm with the ADI for cg06787422 from the EWAS of ADI for the total study population. The dots represent the DNAm beta and ADI values for a participant, and the blue line represents the linear relationship between the DNAm beta values and the ADI. (**H**) Scatterplot of beta values and the ADI from the EWAS of DNAm with the ADI for cg13521319 from the EWAS of ADI for the total study population. The dots represent the DNAm beta and ADI values for a participant, and the blue line represents the linear relationship between the DNAm beta values and the ADI. (**I**) Scatterplot of beta values and the ADI from the EWAS of DNAm with the ADI for cg09431774 from the EWAS of ADI for the total study population. The dots represent the DNAm beta and ADI values for a participant, and the blue line represents the linear relationship between the DNAm beta values and the ADI.
