# Supplementary material for: The association between neighborhood deprivation and DNA methylation in an autopsy cohort
Source: Aging (Albany NY). 2024 Apr 24;16(8):6694–716. doi: 10.18632/aging.205764 (PMC11087100; doi:10.18632/aging.205764)
Supplement: Supplementary Tables 1-3 [file aging-16-205764-s002.pdf]

SUPPLEMENTARY TABLES

Supplementary Table 1. Sample characteristics of the full ADRC cohort.

Sample characteristic<br>n (%) or mean [SD]	Total<br>(n=1011)
**Demographics**	
Race	
White	808 (79.9)
Black	68 (6.7)
Hawaiian	5 (0.5)
American Indian	1 (0.1)
NA	129 (12.8)
Sex	
Male	548 (54.2)
Female	442 (43.7)
NA	21 (2.1)
Age at death	71.3 [13.0]
Education attainment	
High school or less	103 (10.2)
College degree	200 (19.8)
Graduate degree	115 (11.4)
NA	593 (58.7)
**Clinical variables**	
Braak Stage	
Stage 0	86 (8.5)
Stage 1	88 (8.7)
Stage 2	111 (11.0)
Stage 3	70 (6.9)
Stage 4	75 (7.4)
Stage 5	150 (14.8)
Stage 6	298 (29.5)
NA	133 (13.2)
CERAD	
No	294 (29.1)
Sparse	29 (2.9)
Moderate	75 (7.4)
Frequent	542 (53.6)
NA	72 (7.1)
ABC	
Not	161 (15.9)
Low	163 (16.1)
Intermediate	110 (10.9)
High	418 (41.3)
NA	159 (15.7)
APOE ε4 Allele(s)	
0	391 (38.7)
1	318 (31.5)
2	83 (8.2)

NA	219 (21.7)
Cognitive classification	
No dementia	24 (2.4)
Other dementia	569 (56.3)
AD	418 (41.3)

Supplementary Table 2. Top ten CpG sites from the EWAS of DNAm with the Area Deprivation Index (compare Figure 1 and Table 2).

CpG	Chromosome	Position	Gene(s)	Main analysis (n=159)		Adjusted for CERAD (n=159)		Adjusted for ABC (n=159)		Adjusted for braak stage (n=159)	
				Effect estimate	P-value	Effect estimate	P-value	Effect estimate	P-value	Effect estimate	P-value
cg26514961	12	94566784	*PLXNC1*	-0.0052	0.00000005	-0.0050	0.0000000533	-0.0053	0.0000000498	-0.0050	0.0000000533
cg08087060	16	87795808	*KLHDC4*	-0.0040	0.00000057	-0.0038	0.000000592	-0.0041	0.000000562	-0.0038	0.000000592
cg01291468	2	234589374	*UGT1A10; UGT1A7; UGT1A9; UGT1A8*	0.0034	0.0000014	0.0034	0.00000151	0.0035	0.00000138	0.0034	0.00000151
cg05419854	17	19398395	-	-0.0058	0.0000018	-0.0057	0.0000019	-0.0060	0.00000175	-0.0057	0.0000019
cg16241648	7	98923114	*ARPC1A*	0.0016	0.0000021	0.0013	0.00000223	0.0016	0.0000021	0.0013	0.00000223
cg20912923	8	2885516	*CSMD1*	-0.0026	0.0000025	-0.0024	0.00000266	-0.0027	0.00000248	-0.0024	0.00000266
cg15953452	3	63053400	-	-0.0050	0.0000025	-0.0051	0.0000025	-0.0052	0.00000247	-0.0051	0.0000025
cg06787422	15	63331851	-	-0.0024	0.0000031	-0.0025	0.0000031	-0.0025	0.00000309	-0.0025	0.0000031
cg13521319	9	133423844	-	-0.0018	0.0000034	-0.0016	0.00000341	-0.0018	0.0000034	-0.0016	0.00000341
cg09431774	22	25465561	*KIAA1671*	-0.0028	0.0000036	-0.0026	0.00000364	-0.0029	0.0000036	-0.0026	0.00000364

In this sensitivity analysis we additionally adjusted the EWAS of ADI for neuropathology markers (CERAD, ABC, Braak Stage) in separate models.

Supplementary Table 3. Number of mQTLs mapped to the CpGs identified as the top ten CpG sites from the EWAS of ADI.

CpG	Chromosome	Position	# mQTLs identified at p<1e-14 (Min et al.)
cg26514961	12	94566784	82
cg08087060	16	87795808	-
cg01291468	2	234589374	187
cg05419854	17	19398395	-
cg16241648	7	98923114	-
cg20912923	8	2885516	-
cg15953452	3	63053400	-
cg06787422	15	63331851	573
cg13521319	9	133423844	-
cg09431774	22	25465561	-
