# Supplementary material for: The association between neighborhood deprivation and DNA methylation in an autopsy cohort
Source: Aging (Albany NY). 2024 Apr 24;16(8):6694–716. doi: 10.18632/aging.205764 (PMC11087100; doi:10.18632/aging.205764)
Supplement: Supplementary Tables 5-10 [file aging-16-205764-s004.pdf]

Supplementary Table 5A. GO terms that were nominally significant (p<0.05) for the top ten CpG sites from the EWAS of ADI.

	Ontology	Term	N genes in term	N differentially methylated genes	P-value	FDR
GO:0036194	CC	muscle cell projection	3	1	0.000502345421245922	1
GO:0036195	CC	muscle cell projection membrane	3	1	0.000502345421245922	1
GO:0051552	BP	flavone metabolic process	6	1	0.00241426466775728	1
GO:0005885	CC	Arp2/3 protein complex	11	1	0.00354560747835619	1
GO:0052696	BP	flavonoid glucuronidation	9	1	0.00390423191457027	1
GO:0052697	BP	xenobiotic glucuronidation	10	1	0.00411590769484143	1
GO:1902287	BP	semaphorin-plexin signaling pathway involved in axon guidance	10	1	0.00436272584402716	1
GO:0002116	CC	semaphorin receptor complex	9	1	0.00449002681942201	1
GO:0017154	MF	semaphorin receptor activity	10	1	0.00458158214874175	1
GO:1902285	BP	semaphorin-plexin signaling pathway involved in neuron projection guidance	11	1	0.00487691928380841	1
GO:0009812	BP	flavonoid metabolic process	14	1	0.00573835688276425	1
GO:0052695	BP	cellular glucuronidation	17	1	0.00652996834768369	1
GO:0006063	BP	uronic acid metabolic process	22	1	0.00829958662418977	1
GO:0019585	BP	glucuronate metabolic process	22	1	0.00829958662418977	1
GO:0001964	BP	startle response	27	1	0.0105784447630494	1
GO:0015020	MF	glucuronosyltransferase activity	30	1	0.0118114852718722	1
GO:0034314	BP	Arp2/3 complex-mediated actin nucleation	37	1	0.0130563075770592	1
GO:0071526	BP	semaphorin-plexin signaling pathway	37	1	0.0141622932961172	1
GO:0045010	BP	actin nucleation	50	1	0.0177133195926246	1
GO:0035861	CC	site of double-strand break	58	1	0.0194649272443375	1
GO:0042440	BP	pigment metabolic process	66	1	0.0238803078794503	1
GO:0002433	BP	immune response-regulating cell surface receptor signaling pathway involved in phagocytosis	77	1	0.0261103192117255	1
GO:0038096	BP	Fc-gamma receptor signaling pathway involved in phagocytosis	77	1	0.0261103192117255	1
GO:0090734	CC	site of DNA damage	79	1	0.0261634735240811	1
GO:0038094	BP	Fc-gamma receptor signaling pathway	80	1	0.0276743567107917	1
GO:0002431	BP	Fc receptor mediated stimulatory signaling pathway	83	1	0.0281054046566517	1
GO:0050772	BP	positive regulation of axonogenesis	79	1	0.0287765616463737	1
GO:0048013	BP	ephrin receptor signaling pathway	85	1	0.0294559296346882	1
GO:0030838	BP	positive regulation of actin filament polymerization	98	1	0.0349750533314372	1
GO:0050905	BP	neuromuscular process	106	1	0.0387299038011777	1
GO:0006805	BP	xenobiotic metabolic process	112	1	0.0426557934218213	1
GO:0071466	BP	cellular response to xenobiotic stimulus	117	1	0.0441801450153952	1
GO:0032273	BP	positive regulation of protein polymerization	133	1	0.0461578372736143	1
GO:0009410	BP	response to xenobiotic stimulus	124	1	0.0466060117650619	1
GO:0008194	MF	UDP-glycosyltransferase activity	134	1	0.0477513908349988	1

Supplementary Table 5B. Top KEGG pathways for the top ten CpG sites from the EWAS of ADI.

	Term	N genes in term	N differentially methylated genes	P-value	FDR
path:hsa00053	Ascorbate and aldarate metabolism	27	1	0.00935562323527538	0.94649073173396
path:hsa00040	Pentose and glucuronate interconversions	32	1	0.0112598177301082	0.94649073173396
path:hsa00860	Porphyrin metabolism	38	1	0.0136467852899198	0.94649073173396
path:hsa00140	Steroid hormone biosynthesis	58	1	0.0207101214494628	0.94649073173396
path:hsa00830	Retinol metabolism	61	1	0.0224058341419214	0.94649073173396
path:hsa00982	Drug metabolism - cytochrome P450	65	1	0.0233265428658444	0.94649073173396
path:hsa05204	Chemical carcinogenesis - DNA adducts	63	1	0.0239040038630032	0.94649073173396
path:hsa05100	Bacterial invasion of epithelial cells	76	1	0.0266674544457237	0.94649073173396
path:hsa00983	Drug metabolism - other enzymes	76	1	0.0268464761287482	0.94649073173396
path:hsa00980	Metabolism of xenobiotics by cytochrome P450	73	1	0.026888941242442	0.94649073173396
path:hsa04666	Fc gamma R-mediated phagocytosis	94	1	0.0300734939977411	0.950116909157175
path:hsa04976	Bile secretion	86	1	0.0323903491758128	0.950116909157175
path:hsa05135	Yersinia infection	129	1	0.0423779846739126	1

Supplementary Table 6. CpG sites from the EWAS of ADI that were statistically significant in Lawrence et al. (2020).

CpG	Our study		Lawrence et al. (2020)	
	Effect estimate	P-value	Effect estimate	P-value
cg23538773	0.0000271	0.773	0.002352	0.0000000198
cg07390373	0.0000374	0.683	0.001854	0.0000000971
cg18956825	0.0000853185179635161	0.654	-0.00233	0.000000275

Supplementary Table 7. Top ten CpG sites from the EWAS of DNAm with the Area Deprivation Index (compare Figure 1 and Table 2).

CpG	Chromosome	Position	Gene(s)	Main analysis (n=159)		Excluding cognitively normal donors (n=152)	
				Effect estimate	P-value	Effect estimate	P-value
cg26514961	12	94566784	*PLXNC1*	-0.0052	0.00000005	-0.005	0.00000004
cg08087060	16	87795808	*KLHDC4*	-0.004	0.00000057	-0.0039	0.00000055
cg01291468	2	234589374	*UGT1A10; UGT1A7; UGT1A9; UGT1A8*	0.0034	0.0000014	0.0032	0.0000013
cg05419854	17	19398395	-	-0.0058	0.0000018	-0.0056	0.0000014
cg16241648	7	98923114	*ARPC1A*	0.0016	0.0000021	0.0016	0.0000019
cg20912923	8	2885516	*CSMD1*	-0.0026	0.0000025	-0.0024	0.0000023
cg15953452	3	63053400	-	-0.005	0.0000025	-0.0048	0.0000023
cg06787422	15	63331851	-	-0.0024	0.0000031	-0.0024	0.0000029
cg13521319	9	133423844	-	-0.0018	0.0000034	-0.0017	0.0000033
cg09431774	22	25465561	*KIAA1671*	-0.0028	0.0000036	-0.0025	0.0000034

In this sensitivity analysis we excluded the 2.5% cognitively normal donors.

Supplementary Table 8. Sample characteristics of the full ADRC cohort compared to the analysis sample.

Sample characteristic	Total (full cohort)		Samples not included in the analysis		Analysis sample		
n (%) or mean [SD]	(n=1011)		(n=852)		(n=159)		P-Value
**Demographics**							
Race							0.42
White	808	(79.9)	666	(78.2)	142	(89.3)	
Black	68	(6.7)	51	(6.0)	17	(10.7)	
Hawaiian	5	(0.5)	5	(0.1)	0	(0.0)	
American Indian	1	(0.1)	1	(0.0)	0	(0.0)	
NA	129	(12.8)	129	(15.1)	0	(0.0)	
Sex							0.83
Male	548	(54.2)	459	(53.9)	89	(56.0)	
Female	442	(43.7)	372	(43.7)	70	(44.0)	
NA	21	(2.1)	21	(2.5)	0	(0.0)	
Age at death	71.3	[13.0]	69.7	[8.2]	76.6	[10.0]	0.54
Education attainment							0.46
High school or less	103	(10.2)	67	(7.9)	36	(22.6)	
College degree	200	(19.8)	124	(14.6)	76	(47.8)	
Graduate degree	115	(11.4)	68	(8.0)	47	(29.6)	
NA	593	(58.7)	593	(69.6)	0	(0.0)	
**Clinical variables**							
Braak Stage							0.41
Stage 0	86	(8.5)	86	(10.1)	0	(0.0)	
Stage 1	88	(8.7)	72	(8.5)	16	(10.1)	
Stage 2	111	(11.0)	100	(11.7)	11	(6.9)	
Stage 3	70	(6.9)	50	(5.9)	20	(12.6)	
Stage 4	75	(7.4)	58	(6.8)	17	(10.7)	
Stage 5	150	(14.8)	128	(15.0)	22	(13.8)	
Stage 6	298	(29.5)	225	(26.4)	73	(45.9)	
NA	133	(13.2)	133	(15.6)	0	(0.0)	
CERAD							0.64
No	294	(29.1)	259	(30.4)	35	(22.0)	
Sparse	29	(2.9)	25	(2.9)	4	(2.5)	
Moderate	75	(7.4)	65	(7.6)	10	(6.3)	
Frequent	542	(53.6)	432	(50.7)	110	(69.2)	
NA	72	(7.1)	72	(8.5)	0	(0.0)	
ABC							0.67
Not	161	(15.9)	146	(17.1)	15	(9.4)	
Low	163	(16.1)	134	(15.7)	29	(18.2)	
Intermediate	110	(10.9)	88	(10.3)	22	(13.8)	
High	418	(41.3)	325	(38.1)	93	(58.5)	
NA	159	(15.7)	159	(18.7)	0	(0.0)	
APOE ε4 Allele(s)							0.31
0	391	(38.7)	321	(37.7)	70	(44.0)	
1	318	(31.5)	250	(29.3)	68	(42.8)	
2	83	(8.2)	62	(7.3)	21	(13.2)	
NA	219	(21.7)	219	(25.7)	0	(0.0)	

Cognitive classification							0.44
No dementia	24	(2.4)	17	(2.0)	7	(4.4)	
Other dementia	569	(56.3)	503	(59.0)	66	(41.5)	
AD	418	(41.3)	332	(39.0)	86	(54.2)	

Missing values (NA) included for descriptive purpose only; group comparison was done for complete cases.

Supplementary Table 9. Top 25 CpG sites from the EWAS of ADI that were statistically significant for Alzheimer's disease in the Smith et al. (2021) meta-analysis.

CpG	Our study		Smith et al. (2021)	
	Effect estimate	P-value	Effect estimate	P-value
cg12307200	0.0000105	0.90	−0.015	4.48E−16
cg01419713	-0.000139595367796947	0.51	0.022	2.20E−14
cg04874795	0.000082	0.66	−0.022	3.95E−14
cg11823178	0.0000133	0.94	0.016	3.24E−13
cg07061298	-0.0000388	0.77	0.018	4.57E−13
cg13076843	-0.000075	0.52	0.021	7.57E−13
cg25018458	-0.0000111	0.64	0.008	7.87E−13
cg07883124	0.0000109	0.92	0.017	9.10E−13
cg03223072	0.0000524	0.70	−0.014	1.10E−12
cg05066959	0.0000104	0.90	0.024	1.45E−12
cg17881200	-0.00267337	0.02	0.017	1.83E−12
cg19240213	-0.000110791	0.48	0.02	2.29E−12
cg10045881	-0.000110486	0.35	−0.015	2.38E−12
cg02674693	0.00000358	0.98	0.018	3.57E−12
cg06800235	-0.0000166	0.92	−0.017	3.71E−12
cg18264562	-0.000294621	0.12	0.014	5.46E−12
cg01964852	0.0000629	0.58	0.016	5.96E−12
cg01111041	-0.000056	0.11	0.009	6.83E−12
cg15974867	-0.000101198	0.15	0.018	7.46E−12
cg17907520	-0.0000942	0.13	0.011	9.65E−12
cg16988611	-0.0000406	0.69	0.011	9.98E−12
cg13579486	-0.0000288	0.76	−0.012	1.01E−11
cg01681367	0.0000329	0.83	−0.015	1.25E−11
cg01301319	-0.0000516	0.74	0.017	1.54E−11
cg02317313	-0.000024	0.85	0.017	1.69E−11

Supplementary Table 10. Top 10 regions from the regional EWAS of ADI using the dmrff R Package.

Chromosome	Start	End	Effect estimate	P-value (bonferroni-adjusted)
12	18046784	18050274	-0.011	3.93E−4
12	16849738	16858357	0.053	4.27E−4
9	11029484	11038264	-0.044	4.88E−4
7	14048948	14053843	0.062	5.33E−4
2	10394855	10399273	0.083	5.53E−4
8	20483933	20489055	-0.092	8.09E−4
3	12048391	12059282	0.072	8.20E−4
6	32031809	32037018	0.082	8.64E−4
6	30682805	30693326	-0.095	8.94E−4
6	31904765	31909284	-0.102	8.99E−4
